# Noncanonical Compensation of Zygotic X Transcription in Early *Drosophila melanogaster* Development Revealed through Single-Embryo RNA-Seq

**DOI:** 10.1371/journal.pbio.1000590

**Published:** 2011-02-08

**Authors:** Susan E. Lott, Jacqueline E. Villalta, Gary P. Schroth, Shujun Luo, Leath A. Tonkin, Michael B. Eisen

**Affiliations:** 1 Department of Molecular and Cell Biology, University of California, Berkeley, California, United States of America; 2 Howard Hughes Medical Institute, University of California, Berkeley, California, United States of America; 3 Illumina, Hayward, California, United States of America; 4 Vincent J. Coates Genomics Sequencing Laboratory, California Institute for Quantitative Biosciences (QB3), University of California, Berkeley, California, United States of America; Stowers Institute for Medical Research, United States of America

## Abstract

When *Drosophila melanogaster* embryos initiate zygotic transcription around mitotic cycle 10, the dose-sensitive expression of specialized genes on the X chromosome triggers a sex-determination cascade that, among other things, compensates for differences in sex chromosome dose by hypertranscribing the single X chromosome in males. However, there is an approximately 1 hour delay between the onset of zygotic transcription and the establishment of canonical dosage compensation near the end of mitotic cycle 14. During this time, zygotic transcription drives segmentation, cellularization, and other important developmental events. Since many of the genes involved in these processes are on the X chromosome, we wondered whether they are transcribed at higher levels in females and whether this might lead to sex-specific early embryonic patterning. To investigate this possibility, we developed methods to precisely stage, sex, and characterize the transcriptomes of individual embryos. We measured genome-wide mRNA abundance in male and female embryos at eight timepoints, spanning mitotic cycle 10 through late cycle 14, using polymorphisms between parental lines to distinguish maternal and zygotic transcription. We found limited sex-specific zygotic transcription, with a weak tendency for genes on the X to be expressed at higher levels in females. However, transcripts derived from the single X chromosome in males were more abundant that those derived from either X chromosome in females, demonstrating that there is widespread dosage compensation prior to the activation of the canonical MSL-mediated dosage compensation system. Crucially, this new system of early zygotic dosage compensation results in nearly identical transcript levels for key X-linked developmental regulators, including *giant* (*gt*), *brinker* (*brk*), *buttonhead* (*btd*), and *short gastrulation* (*sog*), in male and female embryos.

## Abstract 2

Mmany genes from the X chromosome are expressed at the same level in female and male embryos during early *Drosophila* development, prior to the establishment of MSL-mediated dosage compensation, suggesting the existence of a novel mechanism.

## Author Summary

Variation in gene dose can have profound effects on animal development. Yet every generation, animals must cope with differences in sex chromosome numbers. *Drosophila* compensate for the difference in X chromosome dosage (two in females, one in males) with a mechanism that allows for more transcription of the single X chromosome in males. But this mechanism is not established until over an hour after the embryo begins transcription, during which time a number of important events in development occur such as cellularization and segmentation. Here we use an mRNA sequencing method to characterize gene expression in individual female and male embryos before the onset of the previously characterized dosage compensation system. While we find more transcripts from X chromosomal genes in females, we also find many genes with equal transcript levels in males and females. These results indicate that there is an alternate mechanism to compensate for dosage acting earlier in development, prior to the onset of the previously characterized dosage compensation system.

## Introduction

The earliest stages of animal development are under maternal control until mRNAs deposited prior to fertilization degrade and zygotic transcription is initiated during a period known as the maternal to zygotic transition (MZT). In *Drosophila melanogster*, the MZT occurs amidst the 14 rapid and synchronous mitotic divisions that mark the first several hours of development, with zygotic transcripts appearing as early as mitotic cycle 8 [1]. By cycle 14, when cellularization of the previously syncytial blastoderm occurs, most processes are under the control of zygotic transcripts.

As zygotic transcription begins, the different numbers of X chromosomes (two in females, one in males) results in different transcript levels for a small number of genes on the X chromosome (the X chromosome signal elements, or XSEs), which lead to female-specific expression of the master sex control gene *Sex lethal* (*Sxl*) [2]–[4]. The low levels of SXL in males lead to the male-specific formation of a dosage compensation complex composed of five proteins (MSL-1, MSL-2, MSL-3, MOF, MLE) and two non-coding RNAs (*rox1* and *rox2*) that bind to the X chromosome, hyperacetylate histone H4K16, and induce hypertranscription of the male X chromosome [5]–[8].

However, there is a lag between the onset of zygotic transcription and the establishment of MSL-mediated dosage compensation: the complex is not localized on DNA, and H4K16 acetylation is not detectable, until after the blastoderm stage [9],[10], 60 to 90 min after the onset of zygotic transcription. During this gap, zygotic transcription drives a host of important developmental processes, including segmentation along the anterior-posterior axis, the establishment of tissue layers along the dorsal-ventral axis, and cellularization. These events often require the precise spatial localization and concentration of transcription factors and other proteins. It is therefore interesting that many important blastoderm regulators are on the X chromosome, and thus present in varying dosage in males and females, including the A–P factors *giant* (*gt*), *buttonhead* (*btd*), *orthodenticle* (*otd*) and *runt* (*run*), D–V factors *brinker* (*brk*), *short gastrulation* (*sog*) and *neijire* (*nej*), and the cellularization factor *nullo*.

We were intrigued by the possibility that the absence of MSL-mediated dosage compensation during the MZT might lead to higher levels of mRNAs derived from genes on the X chromosome in females, and sex-specific differences in patterning or cellularization that have not been detected because systematic studies of early developmental transcription have never differentiated male and female embryos.

A variety of approaches have been used to profile zygotic transcription during the MZT, including genome-wide expression profiling with microarrays [11]–[15] and in situ hybridization [16]. However the genomic studies pooled mixed-sex embryos based only on developmental time, and generally have not had sufficient temporal resolution to distinguish events during the rapid mitotic cycles of early development. Embryos produced to lack entire chromosomes or chromosome arms have been used to distinguish maternal and zygotic transcription [12], but the effects of these significant aberrations are unknown. Imaging studies have intrinsically higher temporal resolution, and have used differences in RNA localization to begin to unravel the maternal and zygotic contributions to mRNA pools. But doing such experiments on a genomic scale requires considerable time and resources, and current imaging projects do not distinguish male and female embryos.

To address these limitations, we developed methods to characterize, by sequencing, the mRNA content of individual *D. melanogaster* embryos, which we combined with methods to precisely stage and sex single embryos to generate sex-specific time courses of maternal and zygotic transcript abundance spanning the first wave of early zygotic transcription through the MZT to the end of the blastoderm stage when MSL-mediated dosage compensation is thought to begin [9],[10].

## Results

In order to create a precise time series of zygotic transcription in male and female embryos during embryonic development, we needed methods to demarcate small differences in developmental time, to determine the sex of embryos, and to measure the entire pool of transcripts in these embryos in a way that distinguished mRNAs of maternal and zygotic origin.

### Creating a High-Resolution Time Course

We chose to focus on the period of development bounded by cycle 10 (when early zygotic transcription is detectable) and the completion of cellularization in mitotic cycle 14 (when widespread zygotic transcription has been established, right before MSL-mediated dosage compensation is thought to begin).

**Figure 1:**
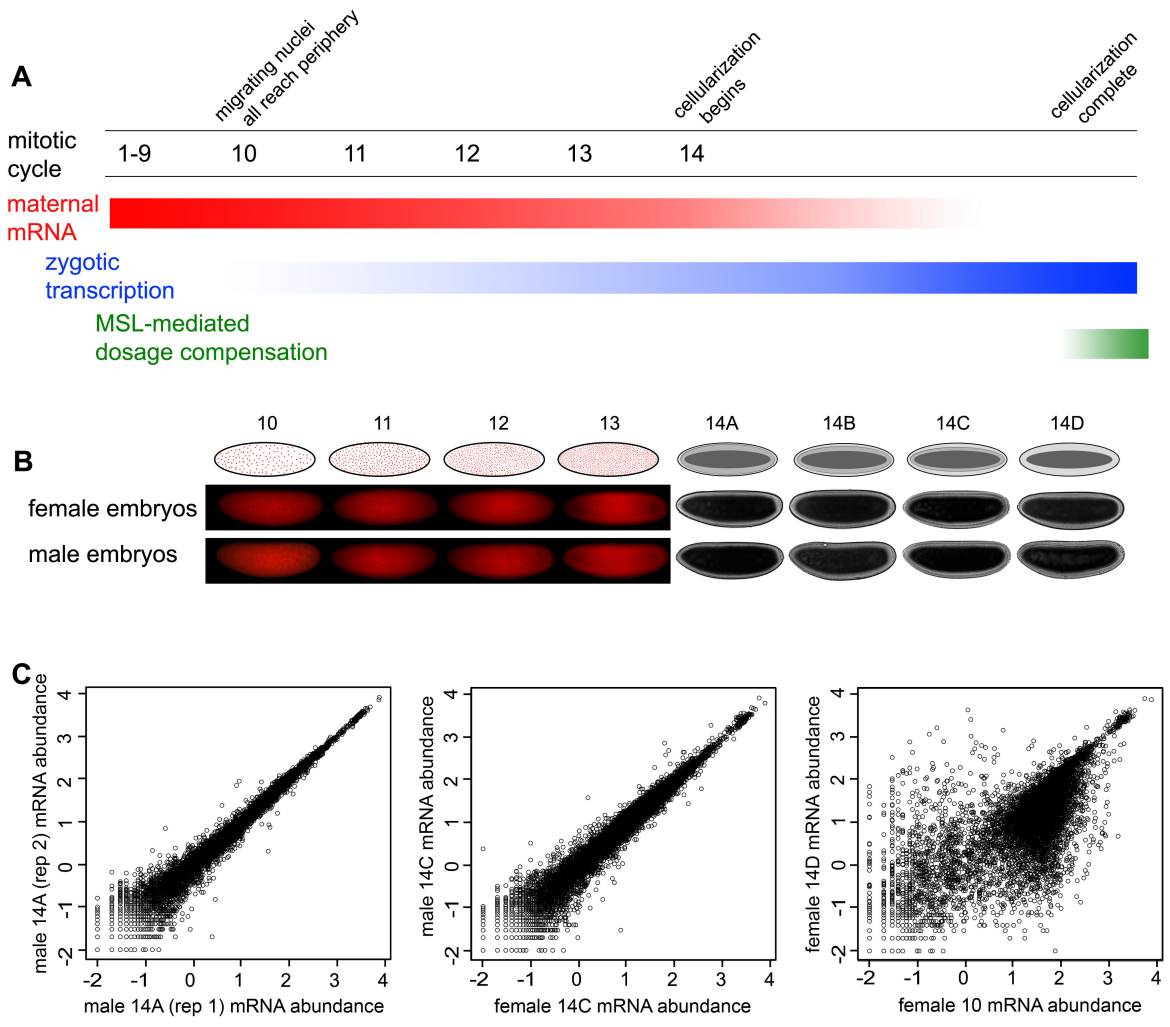
A sex-specific timecourse of early-embryonic gene expression. (A) Transcription events during early embryogenesis. During the first 8–9 mitotic cycles, almost all RNAs in the embryo are of maternal origin. Zygotic transcription begins at a low level at approximately cycle 10 and becomes widespread by the middle of cycle 14. MSL-mediated dosage compensation begins late in or following cycle 14. (B) Embryos used for mRNA-Seq. Individual embryos in the interphases of cycles 10 to 14 were selected by direct observation of mitosis in embryos containing histone H2Av-RFP and computing nuclear density. Embryos at substages of cycle 14 were selected by observing the extent of progression through cellularization (from proportion of membrane invagination) under light microscopy. Each embryo pictured here was placed into TRIzol reagent immediately after these images were taken, DNA and RNA were extracted, and each sample was genotyped to determine the sex of the embryo. (C) Approximately 100 ng total RNA was obtained from each embryo, and poly-A RNA was processed with an amplification-free protocol optimized for small samples and sequenced on an Illumina GAIIx Genome Analyzer. Data (normalized reads per kb, RPKM) from independently processed individuals of the same stage and same sex, and same stage but different sex were extremely similar, while individuals from different stages showed larger numbers of differences.

To determine developmental stage, we took advantage of two characteristics of early embryos: the tightly controlled synchronous mitotic cycles and the process of cellularization as the embryo transitions from a syncytium to a cellular blastoderm (Figure 1). We examined live embryos from a maternal line carrying an RFP-labeled histone under a fluorescent microscope and used a combination of direct observation of mitotic cycles and quantification of nuclear density to select embryos during interphases of mitotic cycles 10, 11, 12, 13, and 14. Stage assignments were based on examination of the entire embryo to avoid cases where different portions were in different mitotic cycles [17]. We further refined the staging within cycle 14 by examining embryos under a light microscope and quantifying the extent of membrane invagination during cellularization, assigning embryos to stages 14A (0%–25% invagination), 14B (25%–50%), 14C (50%–75%), and 14D (75%–100%). Selected embryos were immediately immersed in TRIzol, ruptured, and frozen for subsequent extraction.

We selected at least four embryos each for cycles 10, 11, 12, 13, 14A, 14B, 14C, and 14D, and extracted DNA and RNA from each embryo independently. We carried out whole-genome amplification on the DNA from each embryo and genotyped it for Y chromosomal markers to determine the sex of the embryo, and selected at least one male and female embryo from every stage for transcriptome analysis. Figure 1 shows the embryos we selected immediately before DNA and RNA were extracted.

### Characterizing the Transcriptomes of Single Embryos by RNA-Seq

We obtained 75 to 100 ng of total RNA from each embryo. As this was less starting material than required for standard mRNA sequencing protocols, we modified the Illumina mRNA-Seq protocol to obtain reliable data from such small quantities of input mRNA without amplification by performing all purification and size selection steps using magnetic beads, and reducing the volume of some reactions (a complete protocol is available in Protocol S1). These relatively minor alterations were sufficient to lower the amount of starting material required by more than an order of magnitude.

We sequenced a total of 24 mRNA samples on an Illumina GAIIx Genome Analyzer. We aligned reads to the *D. melanogaster* reference sequence (version 5.23) using Bowtie [18] and inferred transcript levels using TopHat [19] and Cufflinks [20]. We normalized expression levels between samples so that the total inferred expression levels of autosomal transcripts were identical. Statistics on the sequencing and mapping are reported in Table 1.

**Table 1:** Sequencing statistics for single embryo mRNA-Seq samples.

Sex	Stage	Reads	Mapped Reads
Female	10	26,976,249	17,633,235
Female	11	27,988,411	17,105,590
Female	12	23,270,517	19,949,416
Female	13	24,615,381	20,388,812
Female	14A	21,322,865	16,349,815
Female	14A	18,590,935	16,036,761
Female	14B	18,545,524	15,121,569
Female	14B	19,244,061	17,316,317
Female	14C	20,902,589	17,346,048
Female	14C	18,459,025	16,647,754
Female	14D	23,128,318	19,327,028
Female	14D	17,750,907	15,848,586
Male	10	21,892,898	16,468,144
Male	11	18,250,299	12,796,916
Male	12	25,167,764	21,527,094
Male	13	23,830,913	18,156,530
Male	14A	22,696,841	18,738,955
Male	14A	19,301,209	17,208,613
Male	14B	23,409,763	16,417,656
Male	14B	18,501,195	16,617,463
Male	14C	22,471,826	18,740,316
Male	14C	17,617,204	15,340,670
Male	14D	20,536,683	16,674,908
Male	14D	18,532,116	15,888,989

The single embryo mRNA-Seq method was highly reproducible and has a wide dynamic range (Figure 1C). Transcript levels over all genes from individuals of the same sex and stage had correlation coefficients from 0.95–0.97 (Spearman's rank correlation); transcript levels from individuals of the same stage but different sex were correlated to a similar degree. In contrast, transcript levels from embryos of the same sex but different stage had correlations ranging from 0.80–0.97.

### Distinguishing Maternal from Zygotic Transcription Using Polymorphism

In order to distinguish zygotic transcripts from those deposited by the mother, we analyzed embryos produced by a cross of two genetically distinct *D. melanogaster* lines: a *w1* derived maternal line (which contained the His2Av-RFP marker) and a *Canton-S* (*CaS*) paternal line. We sequenced both lines to roughly 35× coverage (see Table 2), mapped reads to the reference genome using maq (maq.sourceforge.net), and identified 285,927 sites that differed between the strains.

**Table 2:** Strain-specific polymorphism statistics from genome sequencing.

	CantonS	w1
Reads	65,907,258	63,716136
Mapped reads	57,375,508	55,489,784
Total mapped bases	5,794,926,308	5,604,468,184
Average coverage	34.3×	33.2×
Sites scored as polymorphic with respect to reference	299,254	340,119
Sites polymorphic between strains	285,927	
Fixed polymorphisms between strains	122,672	

The vast majority of these differences were biallelic single-nucleotide polymorphisms (SNPs) known from resequencing projects to be polymorphic in the North American *D. melanogaster* population (dpgp.org and DGRP). This is consistent with these strains representing independent samples drawn from resident populations in the United States. Although both lines have been in laboratories for decades, we found that each harbored a significant amount of residual polymorphism, especially *CaS*. We therefore restricted our subsequent analyses to a set of 122,672 SNPs that were fixed between strains.

**Figure 2:**
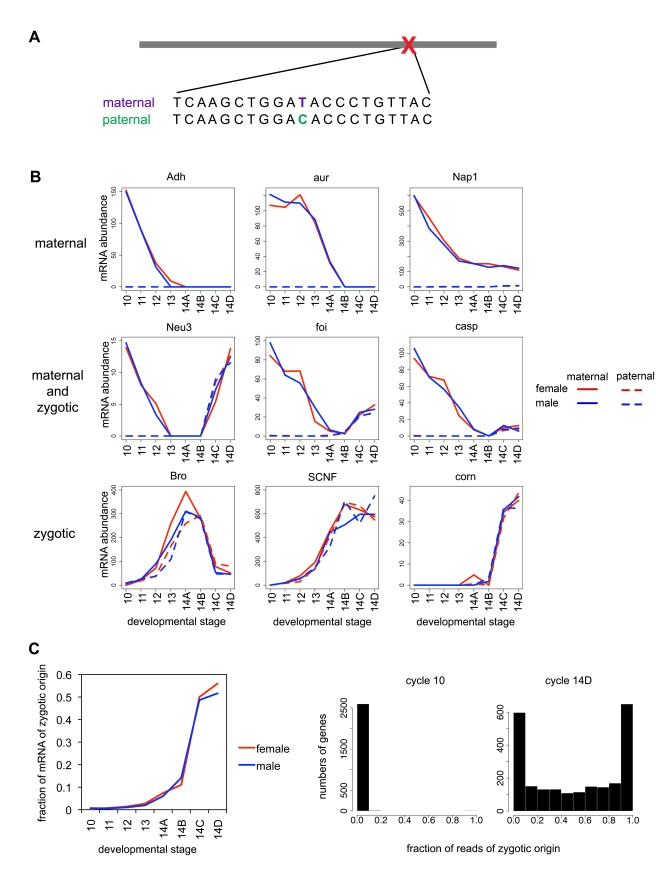
Polymorphisms distinguish maternal and zygotic expression. (A) Approximately 70% of genes expressed in the early embryo contained fixed differences between the maternal (*w1*) and paternal (*CantonS*) lines, allowing us to partition the expression level for that gene at each time point into those derived from the maternal and paternal chromosomes. (B) We classified genes based on temporal profiles of total mRNA and (where available) mRNA derived from maternal and paternal chromosomes. Maternally deposited transcripts (∼5,000) were expressed at high levels that decay over time and come exclusively from the maternal chromosome. Zygotic transcripts (∼2,000) were not present or were present at very low levels at cycle 10, and transcript levels rose over time with equal contribution from maternal and paternal chromosomes. Approximately 800 transcripts are both maternally deposited and zygotically transcribed. (C) Left, the average proportion of zygotic reads per gene increases over time, accelerating during mid cycle 14. Right, a histogram showing the proportion of zygotic reads over genes for an early and a late stage.

Exactly 10,492 of 14,833 annotated genes (over 70%) contained at least one fixed polymorphism, allowing us to assign RNA-Seq reads spanning the polymorphism to either *w1* or *CaS* (Figure 2A). Since maternally deposited mRNAs should all be *w1*, any *CaS* (paternal) reads must have been the result of zygotic transcription. We were thus able to partition the overall expression of any mRNA containing *w1-CaS* differences into its maternal and zygotic component (Figure 2B). As expected, transcripts at cycle 10 were almost entirely maternal (Figure 2C). We observed widespread zygotic transcription beginning in the middle of cycle 14, and by the end of cycle 14, we find a mix of persistent maternal and zygotic transcripts, in varying proportions, depending on the gene (Figure 2C).

We used the strain-specific time series to classify genes as maternal, zygotic, or maternal and zygotic. Briefly, we clustered (k-medians) the 5,226 genes with at least 10 reads spanning a *w1-CaS* polymorphism into 20 groups based on similarity of their inferred abundance of maternally and paternally derived transcripts. We classified each cluster as maternal (only *w1* mRNAs detected with levels declining over time), zygotic (no mRNA at cycle 10, with both *w1* and *CaS* alleles detected over time), or maternal and zygotic (only *w1* mRNAs detected at cycle 10, with *CaS* mRNAs appearing over time). Because of the absence of paternal alleles for genes on the X chromosome in males, all assignments were based on data from females only. We classified genes lacking polymorphisms distinguishing the strains by comparing their mRNA abundances from the eight female samples to the average patterns from each of the previously assigned groups. We assigned genes to the group with which their expression pattern was best correlated (if the correlation coefficient was greater than 0.8). Overall, 5,598 genes were classified as maternal, 2,210 as zygotic, and 1,195 and maternal+zygotic (the classification for each gene is listed in Dataset S1).

### Profiles of Known Sex Determination and Dosage Compensation Factors

**Figure 3:**
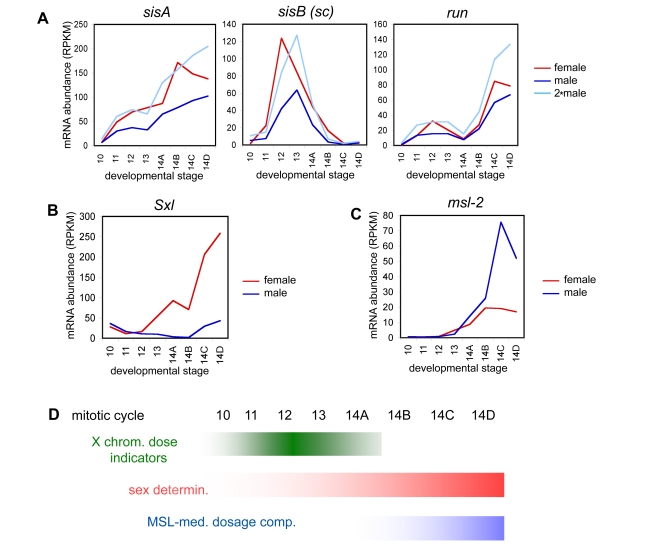
Transcription of sex determination and dosage compensation genes. The events in the sex determination pathway in our data are consistent with previous studies. (A) Expression levels (normalized reads per kb, RPKM) for the X chromosome signal elements (XSEs; *sisA*, *sisB*, and *run*) in female embryos reach twice the transcript abundance of male embryos (light blue line) near cycle 12. These factors activate *Sxl* expression (B) in females, with significant female expression levels around cycle 13, the presence of which interferes with *msl-2* expression (C), the male-specific protein of the MSL-mediated dosage compensation, which is higher in males, starting mid-late cycle 14.

Previous studies of sex determination and dosage compensation have described the expression sex-specific patterns of expression in a number of zygotically transcribed genes [2]–[5]. We examined the expression patterns of these genes in our data to confirm that we could effectively detect transcript differences in zygotically transcribed genes between male and female embryos (Figure 3). As expected, we observed that the numerator genes *sisA*, *sisB* (also known as *sc*), and *run* are expressed at higher levels in females (twice as high during cycles 11–12, Figure 3A), that early *Sxl* expression is substantially higher in females (Figure 3B), and that *msl*-*2* is more abundant in males (Figure 3C). We did not observe *msl-2* transcript until the middle of cycle 14, consistent with earlier studies demonstrating that MSL-mediated dosage compensation is not established until after cellularization [9],[10]. Collectively, these data establish that we can reliably detect sex-specific differences in expression where they exist.

### X Chromosome Transcripts Are Female Biased But Dosage Compensated

**Figure 4:**
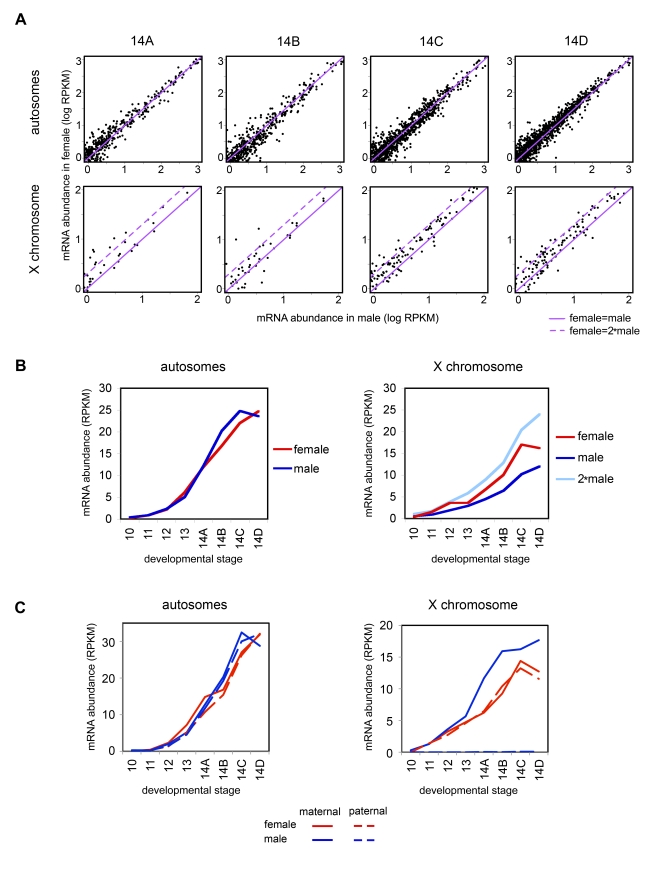
Zygotic transcription from the X chromosome is weakly female biased. (A) Female expression versus male expression for zygotic genes (normalized reads per kb, per gene, log scale) over cycle 14, where most zygotic expression is detected. Autosomal gene expression was centered around the purple line, where female and male transcript levels are even. For X chromosomal genes, transcript levels were distributed between females and males having equal transcript abundance (solid line) and the female having twice the transcript level of the male (dotted line). (B) Total expression levels (average normalized reads per gene) for zygotic genes in male and female embryos, on autosomes and the X chromosome. Female expression on X is less than twice the level of male expression after cycle 12 (light blue line). (C) Zygotic transcripts from autosomal genes were derived equally from the maternal or paternal chromosomes, while zygotic transcripts from the single X chromosome in males are present at higher levels than those from either of the X chromosomes in females, demonstrating that the early embryo is dosage compensated.

We next compared transcript levels of all 2,210 purely zygotic genes in male and female embryos. Zygotically derived transcripts from autosomal genes were observed at the same levels in females and males (Figure 4A). In contrast, zygotically derived transcripts from the X chromosome were consistently observed at higher levels in females than in males (Figure 4A), with a female to male ratio ranging from 1.0 to 2.0. The female to male ratio, and thus the level of compensation, did not correlate with expression level of the gene, or the position of the gene on the X chromosome.

The difference between the X chromosome and autosomes can be seen clearly when total abundance of zygotically expressed genes in males and females is compared between the X chromosome and autosomes (Figure 4B). Autosomal transcript levels were effectively identical in females and males at all time points, and X chromosome transcript levels were higher in females, yet not twice as high as in males. The ratio of transcript levels of zygotic genes from the female to male X chromosomes was approximately 1.45 over cycle 14 (mean of 1.5, median of 1.4, over all X chromosomal zygotic genes; for zygotic genes on chromosome 2L, mean and median female to male ratios are 1.1 and 1.0, respectively).

We observed no difference in the levels of transcripts derived from maternal or paternal chromosomes for either autosomes or (in females) the X chromosome. Total expression of zygotic genes from the paternal and maternal X chromosomes of females was very similar (average Spearman's rank correlation ρ = 0.97 across stages, some as high as ρ = 0.999; Figure 4C). The total abundance of zygotic genes from the single male X chromosome was consistently higher (Figure 4C) than from either female X chromosome—demonstrating that transcript abundance in the early embryo transcription is subject to some form of dosage compensation.

### Transcript Levels of Key Developmental Regulators on the X Chromosome Are Nearly Completely Compensated

**Figure 5:**
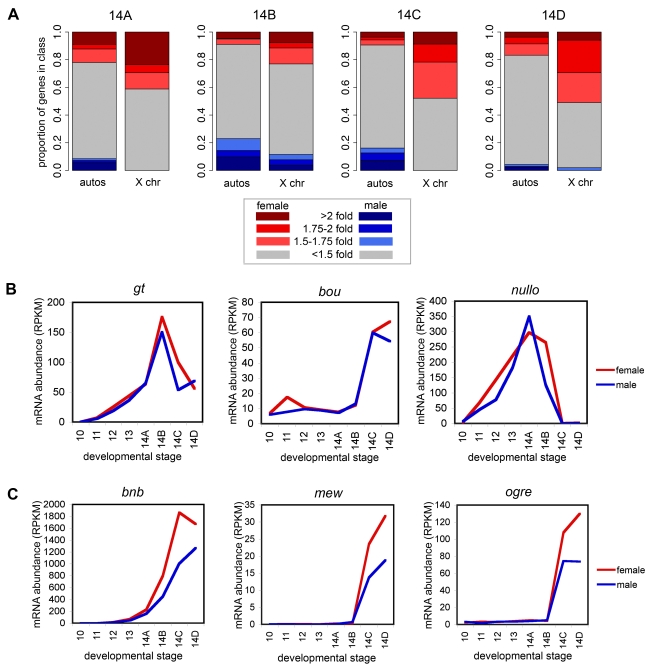
Early zygotic dosage compensation. Of the zygotic genes on the X chromosome, some had the same transcript levels in female and male embryos, and some had an excess of transcripts in females relative to the male, indicating that some are transcriptionally dosage compensated and some are not. (A) Proportion of genes that had higher transcript levels in males or females over cycle 14, comparing autosomes to the X chromosome. The darker colors represent a stronger enrichment of female or male transcripts relative to the other sex. To reduce noise, ratios of female to male expression were considered for genes where individuals of both sexes had at least 2 RPKM, little qualitative difference was observed in results for higher thresholds (results not shown). (B) Key developmental regulators on the X chromosome were dosage compensated at the transcript level. (C) Other zygotic factors on the X did not appear to be effectively dosage compensated, as there were large differences in expression between male and female embryos.

The sex ratio of transcript abundance for individual genes varied somewhat over cycle 14, especially earlier in cycle 14 where there are not many zygotic genes expressed. But across cycle 14, the X chromosome consistently had an excess of genes with higher transcript level in females (Figure 5A). Yet by the end of cycle 14, there were few genes on the X chromosome that had a 2-fold excess of transcript levels in females, and only approximately 30% had more than a 1.75-fold enrichment. Approximately half of the factors on the X chromosome had less than a 1.5-fold excess of transcripts in females.

The expression patterns of the patterning genes whose presence on the X chromosome motivated us to examine sex-specific expression in the early embryo were particularly striking. For example, *giant*, a textbook example of an important early embryonic regulator on X for which differences in levels would likely impact development [21], was almost perfectly dosage compensated (Figure 5B), with equal transcript levels in males and females corresponding to roughly 2-fold greater abundance of mRNAs derived from the paternal X chromosome compared to the X chromosomes in females. Other key X-linked developmental regulators, including *vnd*, *nullo*, *btd*, *tsg*, and *sog*, were also present at roughly equal levels in males and females.

As is often the case with dosage compensation mechanisms, early zygotic dosage compensation is not universal, and several genes showed no evidence of compensation at the transcript level (Figure 5C). We assigned every zygotic gene with appreciable expression levels (maximum normalized RPKM greater than 3.0) a compensation score equal to the slope of the line fit (by least squares) to the male and female transcript levels for that gene. The distribution of these values for autosomal genes were centered around 1.0 and rarely showed a greater than 1.5-fold difference (Figure S1A). In contrast, 77 of the 85 zygotic genes on the X chromosome were greater than 1.0 and 36 were greater than 1.5 (Figure S1A). These compensation scores are given in Dataset S1, and plots of all zygotic genes on X sorted by this score are shown in Figure S1B.

## Discussion

### Mechanisms of Early Zygotic Dosage Compensation

Our development of methods to examine sex-specific gene expression in early *D. melanogaster* embryos was motivated by the expectation that the earliest stages of zygotic transcription are not dosage compensated and that resultant sex differences in the levels of crucial patterning genes might have interesting phenotypic consequences.

Instead, our genome-wide time course of transcript levels in individual male and female embryos has revealed extensive dosage compensation of X chromosomal transcript levels before the canonical MSL-mediated dosage compensation process is thought to be engaged. Crucially, mRNAs for key X-linked developmental regulators, including *gt*, *brk*, *btd*, and *sog*, are present at essentially identical levels in male and female embryos.

Although there is clearly early zygotic dosage compensation (EZDC), our data speak only indirectly to the mechanism by which it occurs. Assuming that, in an uncompensated system, we would expect transcription to produce twice as many zygotically derived copies of X chromosomal genes in females than in males, the generally lower levels we observe in females must arise through sex and X-chromosome-specific transcriptional or post-transcriptional regulation.

The simplest explanation is that the MSL-based dosage compensation system is active before and during cycle 14, leading to hypertranscription of the male X. However, several imaging studies of the male-specific localization of MSL proteins to, and the subsequent acetylation of histones on, the male X chromosome describe an at least 1 h lag between the onset of zygotic transcription and these hallmarks of MSL-mediated dosage compensation [9],[10]. While it is possible that these studies missed earlier low-level or highly targeted MSL-binding and compensation that escaped detection in the microscope, independent evidence exists for MSL-independent dosage compensation in the early embryo [22].

Through an analysis of larval cuticle patterns of male and female embryos carrying various combinations of *run* hypomorphic alleles, Gergen demonstrated that the X-linked gene *run*, which is involved in both sex-determination and segmentation, is functionally dosage compensated [22]. Although *run* is expressed throughout embryogenesis, the effects on larval cuticle patterns these studies examined arise during the blastoderm stage and are thus an example of EZDC. We also find that *run* is dosage compensated during cycle 14. Gergen [22], and later Bernstein and Cline [23], showed that dosage compensation of *run* is MSL independent but requires the early female-specific form of *Sxl*.

Since SXL is an RNA-binding protein known to modulate splicing and translation, it was proposed that dosage compensation of *run* might result from direct SXL-mediated reduction of the translation or stability of *run* in females [24]. Consistent with this possibility, the 3′UTR of *run* mRNA contains several matches to the SXL consensus sequence [24]. However, a direct role for SXL in *run* dosage compensation has not been confirmed.

The two best-characterized targets of SXL are *msl*-*2* mRNA, which it regulates by translational repression, and its own mRNA, which it regulates by controlling how it is spliced. However, a total of 88 genes (including *run*) have transcripts whose 3′UTRs contain three or more SXL target sites (AUUUUUUU or UUUUUUUU). And of these an astonishing 76 are on the X chromosome. This striking enrichment, originally noted by Kelley et al. [24] and expanded by Cline [25], suggests a broad role for SXL in specifically regulating the stability or activity of mRNAs derived from the X chromosome. If the female-specific SXL is controlling EZDC directly, it would have to do so by reducing the levels of X chromosomal RNAs in females, as SXL is not present in males. While such an activity has not been established for SXL, many other RNA binding proteins are known to affect transcript levels [26]–[29].

There is, however, imperfect agreement between predicted SXL targets and genes we observe to be dosage compensated. Many genes with high degrees of EZDC are not predicted SXL targets (Figure S1; *gt*, for example, is not a predicted target) and many predicted SXL targets are not or are poorly dosage compensated (Figure S1B). Furthermore, many predicted SXL targets on X are maternally deposited, with no early zygotic transcription. These genes are not expected to be affected by chromosomal dosage differences. Indeed SXL acting to reduce the levels of these genes in females would produce, rather than eliminate, dosage differences. To resolve whether SXL plays a role in EZDC, we are currently determining whether EZDC is present in *Sxl* mutants, and whether SXL interacts specifically with EZDC targets.

If it turns out that neither the MSL complex or *Sxl* are required, it is possible that dosage compensation arises from gene-specific feedback. Many developmental regulators regulate their own transcription [30],[31], and such interactions could lead to full or partial compensation of initially higher transcript levels in females than males. However, this kind of feedback would also likely have a significant time lag between the emergence of differences in transcript levels and their compensation. There is evidence that the early embryo is generally robust to environmental factors such as temperature and some forms of genetic variation [32]–[35]. Systems conferring such robustness might also sense and compensate for deviations arising from differences in X chromosomal dose.

Each of the models discussed above assume that, without intervention, 2-fold differences in DNA dose inherently produce 2-fold differences in transcription and transcript abundance, which need, at least for some subset of genes, to be compensated. However, this is not necessarily the case. Studies on autosomal regions with altered dosage in *Drosophila* suggest an average 1.3–1.5-fold increase in transcript level per copy [36]–[38]. Dosage compensation of the X chromosome in *Drosophila* results in a ∼2-fold increase in transcription in males, relative to the autosomes [36]–[38]. A recent study [39] estimates that the MSL-complex has a 1.35-fold effect on expression of the X chromosome in males, and suggests that X chromosome dosage compensation could simply be the interaction of this 1.35× effect with the baseline 1.5× dosage effect. However, the effects of these altered gene dosages in these experiments, which measure precise differences in expression, are unknown. It is unclear whether these dosage differences are comparable to the wild-type differences in X chromosome dosage, and how to interpret the quantitative effects as characterized. Regardless of what the baseline threshold for compensated versus uncompensated transcription is with a 2-fold dosage difference, we see many factors on the X chromosome with no difference in transcription rates in males and females.

Additionally, the expectations of the interactions of gene dosage and expression may not be the same in the unique transcription environment of the early embryo. A recent study by Lu et al. [40] compared gene expression during early development in diploid and haploid embryos and found that transcript levels for a large class of zygotically transcribed genes (those whose transcription is dependent on developmental time, rather than nucleocytoplasmic ratio) were dosage independent.

To explain this observation, Lu et al. [40] proposed a model in which transcription is limited by an unknown, maternally deposited, factor. Since both haploid and diploid embryos would have the same amount of this limiting factor, and since individual genes would be present in the same proportion to each other, rates of transcription across the genome would be the same. However, the limiting factor hypothesis cannot explain X chromosomal dosage compensation, as halving the dosage of X chromosomal genes relative to autosomal genes in males would lower the relative rate of transcription of X chromosomal genes (compared to autosomes) at any concentration of the limiting factor.

There is a related alternative to the limiting factor hypothesis that could explain both dosage compensation and insensitivity to ploidy, concerning the accessibility of DNA templates. Homologous chromosomes are known to be paired throughout *Drosophila* development [41],[42], and imaging of nascent transcripts in the early embryo consistently shows the close proximity of transcribed alleles. Given that transcription involves localization to specific subnuclear regions and attachment to large protein machines, it seems possible that the transcription of one allele could make it difficult or even impossible to transcribe the other allele. If such an effect occurred, then the embryo will be inherently dosage compensated. If only one copy of a gene is present (for the whole genome in haploid embryos or the X chromosome in males), it is transcribed at whatever rate the various regulatory systems active dictate. If two copies of the same gene are present (as in diploids and females), the gene would be expressed at the haploid level, with expression divided across the two alleles.

While no such mechanism has been described, the rapid mitotic cycles of early development place constraints on transcription [43] and might make the early embryo particularly sensitive to such effects. It has also long been observed in Diptera, that homologous chromosomes pair during mitosis, as well as meiosis [41],[42]. Expression can be affected by the pairing of homologs, through phenomena such as transvection [44]–[46], the control of genes by regulatory interactions with their homologs in *trans*. Pairing of some homologous loci is observed as early as cycle 13 and increases through cycle 14 [47]–[50], precisely at the times EZDC is observed. As pairing of homologous loci also seems to occur at particular sites rather than “zippering” along a chromosome [48], this could also explain why some sites seem compensated and others do not.

Yet, contrary to this, near synchronous appearance of two adjacent dots in many nuclei in RNA in situ hybridization of intronic probes from autosomal genes demonstrates that paired alleles can both be transcribed at roughly the same time [4],[51],[52]. But it leaves open the possibility that the transcription of one allele could affect the rate at which the other is transcribed.

Whatever the mechanism turns out to be, our data provide an unprecedented window on the temporal dynamics of transcript levels in male and female embryos, and establish that some mechanism exists that ensures that differences in sex chromosome dose do not translate into differences in mRNA abundance during a crucial period of *D. melanogaster* development.

### Beyond Dosage Compensation

While our focus here was on dosage compensation, our data represent a significant advance over earlier methods to monitor gene expression in the early *D. melanogaster* embryo by providing higher temporal resolution and precision, sex specificity, and unambiguous discrimination of maternally deposited and zygotically transcribed mRNAs. Our use of individual embryos also provides a window onto embryo-to-embryo variability in transcript levels, which we found to be surprisingly low.

We hope that our data, which are being made available in full here, will help address a number of other open questions about transcription during early *D. melanogaster* embryogenesis. And we suspect that the methods we developed for analyzing mRNA from individual *Drosophila* embryos and other aspects of our experimental design will be of interest to researchers interested in the analysis of small RNA samples. Although our experiments worked exceptionally well, in carrying them out, we made several observations that should be of interest and use to other investigators.

First, we routinely obtained at least 10 times more material from processing the RNA from a single embryo than was needed for a single Illumina sequencing lane. This suggests that the RNA content of even smaller samples could be routinely analyzed without RNA amplification. Second, for a variety of reasons, mostly involving cost, we carried out 36 base pair single-end sequencing runs. In retrospect, we would have been able to assign many more reads to distinct parental chromosomes, and perhaps detected sex-specific splicing, had we carried out longer, paired-end runs. Finally, analyzing embryos from a cross of divergent strains was very useful. But we were surprised at how polymorphic the supposedly inbred strains we used in our crosses were. We suspect this is a general phenomenon, and suggest that all researchers doing experiments that require highly inbred lines specifically inbreed the lines they are using and resequence them to characterize residual polymorphism prior to use.

## Materials and Methods

### Fly Line and Imaging

Flies were raised on standard fly media in uncrowded conditions, at room temperature. 2–3-d-old virgin females of the *His2Av-mRFP1* III.1 line (Bloomington stock center, stock no. 23650) [53] were crossed to *Canton-S* males, and eggs were collected from many 3–6-d-old females, thus minimizing chances that multiple embryos sampled would come from the same mother. After collection, eggs were dechorionated, placed on a slide in halocarbon oil, and visualized using a Nikon Eclipse 80i microscope, with a Nikon DS-UI camera, and the NIS Elements F 2.20 software. Embryos were photographed both for fluorescence with an RFP filter to visualize nuclei and under white light with a DIC filter to visualize the extent of cellularization in mitotic cycle 14 embryos (Figure 1B). Embryos were then moved from the slide, cleaned from excess oil, and placed in a drop of TRIzol reagent (Invitrogen) within a minute or less of imaging. Embryos are then ruptured with a needle, allowed to dissolve, and moved to a tube with more TRIzol reagent, which was then frozen at −80°C. For determining the age (mitotic cycle) of each embryo, images of nuclei were analyzed in ImageJ (1.42q), where nuclear numbers were counted in a 200×200 pixel box, to confirm the nuclear division cycle of each embryo. For those embryos within mitotic cycle 14, the DIC photographs showing the extent of membrane invagination were used to create subclasses within cycle 14 (Figure 1B).

### RNA Extraction, Genotyping, and Sequencing Library Preparation

RNA and DNA extraction from single embryos was done with TRIzol (Invitrogen) reagent according to the manufacturer's protocol, except with a higher volume of reagent relative to the amount of material (i.e. starting with 1 mL of TRIzol despite expecting very small amounts of DNA and RNA). Extracted DNA was amplified using the Illustra GenomiPhi V2 DNA Amplification Kit (GE Healthcare), and embryos were sexed by detecting the presence of a Y chromosome, using PCR with primers to a region of the male fertility factor kl5 on the Y chromosome (forward primer GCTGCCGAGCGACAGAAAATAATGACT, reverse primer CAACGATCTGTGAGTGGCGTGATTACA), and a region on chromosome 2R (forward primer AAAAGGTACCCGCAATATAACCCAATAATTT, reverse primer GTCCCAGTTACGGTTCGGGTTCCATTGT) as a control.

Total RNA was made into libraries for sequencing using the mRNA-Seq Sample Preparation Kit from Illumina, following an altered mRNA-Seq library making protocol developed at Illumina (see complete protocol in Protocol S1). Libraries were quantified using the Kapa Library Quantification Kit for the Illumina Genome Analyzer platform (Kapa Biosystems), on a Roche LC480 RT-PCR machine, according to the manufacturer's instructions.

### RNA Sequencing

An alternate flow cell loading protocol for small concentration sequencing libraries was developed for this study and used here, despite the libraries created largely being concentrated enough not to necessitate use of this method (see Protocol S2). For each sample, 40 pM of library (relative to final concentration loaded on to flow cell) was diluted in 4 uL, and 1 uL of 0.5 M sodium hydroxide was added. Samples were left 5 min to denature, then placed on ice, and 1 uL 0.5 M hydrochloric acid added, then diluted to final loading concentration (of at least 20 uL) with Illumina hybridization buffer. To load sample on an Illumina flow cell, an air gap was created, the entire sample drawn into the hybridization manifold, an air gap left after the sample, and hybridization buffer used to push the sample until it is centered on the flow cell (see complete protocol in Protocol S2). The rest of the cluster generation and sequencing were according to normal protocols, for 40 cycle sequencing with the Illumina Genome Analyzer (GAIIx).

### Genome Sequencing

We prepared genomic DNA from 10 females from our *CaS* and *w1* stocks. We prepared Illumina paired-end sequencing libraries using standard protocols and sequenced two 101 bp paired-end lanes for each strain on an Illumina GAIIx Genome Analyzer.

### Data, SNP Detection, Mapping, Calling Maternal and Zygotic

Reads from each RNA-Seq sample were mapped to the reference *D. melanogaster* genome (FlyBase release 5.27 [54],[55]) using Bowtie [18] and TopHat [19], and transcript abundances for annotated RNAs were called by Cufflinks [20]. Data from each sample were normalized so that the total expression (reads per kb of sequence, per million mapped reads; RPKM) of autosomal genes was constant. Genomic reads were mapped to the *D. melanogaster* genome (FlyBase release 5.27) using maq (maq.sourceforge.net). We found that consensus base and SNP calling algorithm was adversely affected by the high level of polymorphism, especially in the *CaS* sample, so we exported the base-by-base pileup from maq and developed our own SNP calling algorithm. We designated a position as a *CaS-w1* SNP if there were at least 13 reads covering the base in each strain, if the frequency of the most common base in each strain was at least 95%, and if these most frequent bases differed. We also generated a *w1-CaS* consensus sequence consisting of the reference *D. melanogaster* bases, except where the sequences of the two strains agreed but differed from the reference. We identified all RNA reads expected to differ between the strains, counted their frequencies in each sample, and partitioned the RPKM values for individual genes into their *w1* and *CaS* components in proportion to the fraction of reads in that sample that mapped to the maternal or paternal chromosome. Upon examination of the data, we became concerned that absence of reads from the paternal X chromosome and the low levels of Sxl in embryo F13 arose from a genotyping error. So for graphs showing single genes, we use an average between F12 and F14A for this time point.

We used the strain-specific time series to classify genes as maternal, zygotic, or maternal and zygotic. We clustered (k-medians) the 5,226 genes with at least 10 reads spanning a *w1-CaS* polymorphism into 20 groups based on similarity of their inferred abundance of maternally and paternally derived transcripts using Cluster 3.0 [56]. We classified each cluster as maternal (only *w1* mRNAs detected with levels declining over time), zygotic (no mRNA at cycle 10, with both *w1* and *CaS* alleles detected over time), or maternal and zygotic (only *w1* mRNAs detected at cycle 10, with *CaS* mRNAs appearing over time). Because of the absence of paternal alleles for genes on the X chromosome, all assignments were based on data from females only. We classified genes lacking polymorphisms distinguishing the strains by comparing their mRNA abundances from the eight female samples to the average patterns from each of the previously assigned groups. We assigned genes to the group with which their expression pattern was best correlated (if the correlation coefficient was greater than 0.8).

### Data Availability

All reads have been deposited in the NCBI GEO under the accession number GSE25180 and will be made available at the time of publication. The processed data are available at the journal website (Dataset S1) and at eisenlab.org/dosage.

## Supporting Information

Figure S1 Extent of dosage compensation for zygotic genes on the X chromosome. Each gene was assigned a female to male (F∶M) ratio score equal to the slope of the line fit (by least squares) to the male and female transcript levels for that gene over all time points. (A) Proportion of genes with F∶M ratios between 1.0 (equal expression in males and females) and 2.0 (twice expression in females). (B) Transcript abundance time series for zygotic genes on the X chromosome, in female and male embryos, over all time points. Genes sorted by F∶M ratio.
(2.11 MB PDF)

Dataset S1 Normalized read counts per gene for each individual embryo.
(2.89 MB TXT)

Protocol S1 Small sample RNA-Seq protocol.
(0.14 MB PDF)

Protocol S2 Low concentration sequencing library loading protocol.
(0.08 MB PDF)

## Abbreviations

*brk*: *brinker*
*btd*: *buttonhead*
EZDC: early zygotic dosage compensation
*gt*: *giant*
MZT: maternal to zygotic transition
*nej*: *neijire*
*otd*: *orthodenticle*
*run*: *runt*
SNPs: single-nucleotide polymorphisms
*sog*: *short gastrulation*
*Sxl*: *Sex lethal*
*tsg*: *twisted gastrulation*
*vnd*: *ventral nervous system defective*
XSEs: X chromosome signal elements
