# Supplementary material for: Noncanonical Compensation of Zygotic X Transcription in Early Drosophila melanogaster Development Revealed through Single-Embryo RNA-Seq
Source: PLoS Biol. 2011 Feb 8;9(2):e1000590. doi: 10.1371/journal.pbio.1000590 (PMC3035605; doi:10.1371/journal.pbio.1000590)
Supplement: Figure S1 — Extent of dosage compensation for zygotic genes on the X chromosome. Each gene was assigned a female to male (F∶M) ratio score equal to the slope of the line fit (by least squares) to the male and female transcript levels for that gene over all time points. (A) Proportion of genes with F∶M ratios between 1.0 (equal expression in males and females) and 2.0 (twice expression in females). (B) Transcript abundance time series for zygotic genes on the X chromosome, in female and male embryos, over all time points. Genes sorted by F∶M ratio. (2.11 MB PDF) [file pbio.1000590.s001.pdf]

A

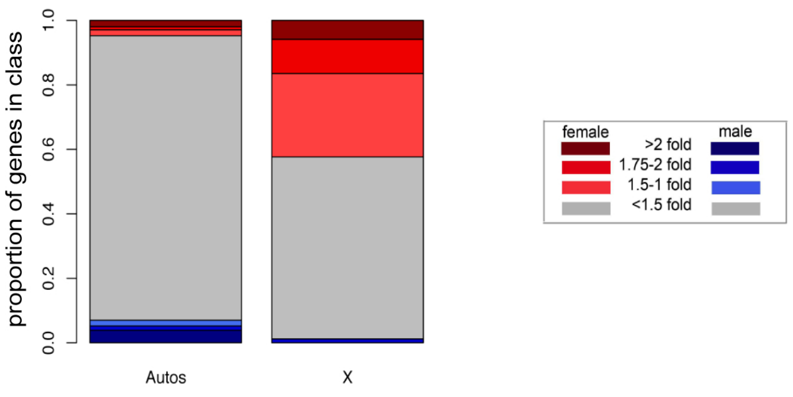
proportion of genes in class
1.0
0.8
0.6
0.4
0.2
0.0
Autos
X
female
male
>2 fold
1.75-2 fold
1.5-1 fold
<1.5 fold

B

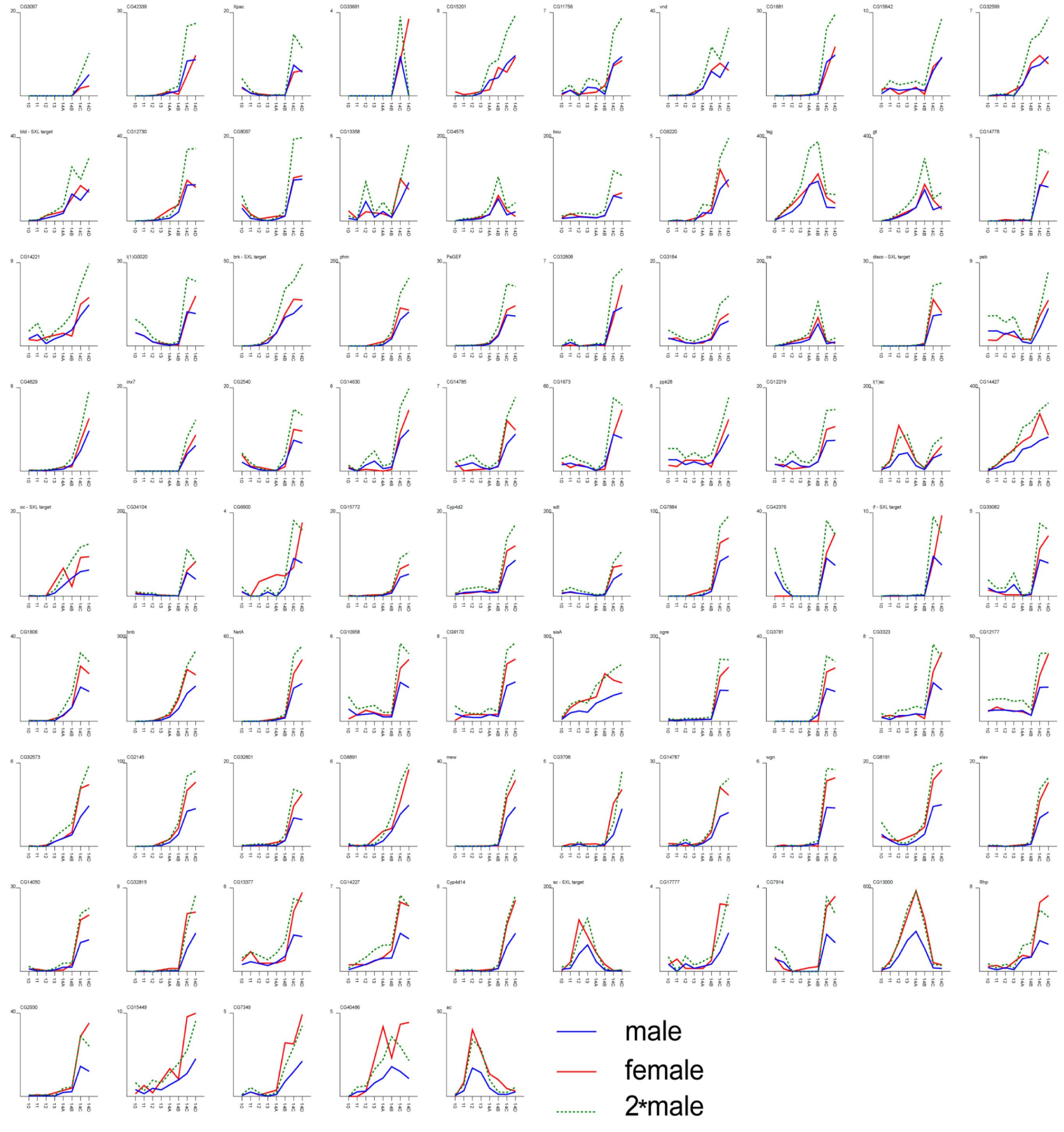
male
female
2*male

Supplemental Figure 1. Extent of dosage compensation for zygotic genes on the X chromosome. Each gene was assigned a female to male (F:M) ratio score equal to the slope of the line fit (by least squares) to the male and female transcript levels for that gene over all timepoints. A) Proportion of genes with F:M ratios between 1.0 (equal expression in males and females) and 2.0 (twice expression in females) B) Transcript abundance timeseries for zygotic genes on the X chromosome, in female and male embryos, over all timepoints. Genes sorted by F:M ratio.
