# Supplementary material for: Noncanonical Compensation of Zygotic X Transcription in Early Drosophila melanogaster Development Revealed through Single-Embryo RNA-Seq
Source: PLoS Biol. 2011 Feb 8;9(2):e1000590. doi: 10.1371/journal.pbio.1000590 (PMC3035605; doi:10.1371/journal.pbio.1000590)
Supplement: Protocol S1 — Small sample RNA-Seq protocol. (0.14 MB PDF) [file pbio.1000590.s003.pdf]

1. **mRNA purification** (you will need two heat blocks, one at 80^0C and one at 65°C. Vortex beads vigorously in each step to resuspend and wash, do not allow beads to dry. The Illumina mRNA-Seq sample prep guide has more guidance as to how to work with the beads.)

   1.1 Dilute >80 ng total RNA with nuclease-free H_2O to 50µL in a 1.5 mL RNase free non-stick tube.
   1.2 Heat at 65°C for 5 minutes to disrupt the secondary structures, and place on ice.
   1.3 Meanwhile, vortex well and aliquot 15µL of Sera-mag oligo(dT) beads into a 1.5mL RNase free non-stick tube.
   1.4 Wash the beads twice with 100µL Bead Binding Buffer.
   1.5 Resuspend the beads in 50µL Bead Binding Buffer, and add the 50µL of total RNA sample from step 1.2; rotate at RT for 5 minutes.
   1.6 Remove the supernatant and wash the beads twice with 200µL of Bead Washing Buffer. Vortex well to re-suspend beads.
   1.7 Add 50µL of 10mM Tris-HCl to the beads, heat at 80^0C for 2 minutes to elute mRNA from the beads
   1.8 Meanwhile aliquot 50µL of Bead Binding Buffer to a fresh 1.5mL RNase free non-sticky tube.
   1.9 After heating the bead and mRNA at 80^0C for 2 minutes, immediately put on the magnet stand and transfer the supernatant (mRNA) to the binding buffer tube from step 1.8.
   1.10 Heat the samples in the binding buffer at 65°C for 5 minutes to disrupt the secondary structures, and place on ice.
   1.11 Meanwhile Wash the bead twice with 200µL of Bead Washing Buffer(Vortex Well)
   1.12 Add 100µL of mRNA sample from step 1.9 to bead and vortex to resuspend beads; rotate at RT for 5 minutes.
   1.13 Remove the supernatant and wash the beads twice with 200µL of Bead Washing Buffer. Vortex well to resuspend beads.
   1.14 Add 17µL of 10mM Tris-HCl to the beads, heat at 80^0C for 2 minutes to elute mRNA from the beads. Immediately put on the magnet stand and transfer the supernatant (mRNA) to a fresh 200µL thin wall PCR tube, and there should be ~16µL of mRNA.

2. Fragment mRNA

2.1 Assemble the following reaction:

- 5x fragmentation buffer 4µL
- mRNA 16µL

2.2 Incubate the tube in a PCR thermocycler at 94°C for 12 minutes, put the tubes on ice and add 2µL of fragmentation stop solution.

2.3 Add 80 µL of 100% Ethanol to fragmented RNA sample, then transfer RNA/Ethanol mix to a 1.5 mL tube containing 60 µL of SPRI beads. Elute the RNA in 12 µL of EB. Perform SPRI purification by following "**SPRI purification protocol"** shown at the end of this protocol.

3. First strand cDNA synthesis

3.1 Assemble the following reaction:

- Random Primer (3ug/µL) 1 µL
- mRNA 11.1µL

3.2 Incubate the tube in a PCR thermocycler at 65°C for 5 minutes, and put the tubes on ice.

3.3 Mix the following in order, make 10% extra reagent for multiple samples:

- 5× 1st strand buffer 4µL
- 100mM DTT 2µL
- dNTP mix (25mM) 0.4µL
- RNAseOUT (40U/µL) 0.5µL

3.4 Add 6.9µL mixture to the tube, mix well, and heat the sample at 25°C in a thermocycler for 2 min.

3.5 Add 1µL SuperscriptIII (200U/ µL) to the sample, and incubate the sample in a thermocycler with following program:

- Step 1 25°C 10min
- Step 2 50°C 50min
- Step 3 70°C 15min
- Step 4 4 °C Hold

4. Second strand cDNA synthesis

4.1 Put the tubes on ice.

4.2 Add 18μL of H_2O to the first strand cDNA synthesis mix.

4.3 Add the following reagents:

- 10 × second strand buffer 5μL
- dNTP mix (25mM) 1μL

4.4 Mix well, incubate on ice 5 minutes and add:

- RNaseH (2U/μL) 1μL
- DNA pol I (10U/μL) 5μL

4.5 Mix well, and incubate at 16°C in a thermocycler for 2.5 hours.

4.6 Purify the DNA by adding 75 uL of SPRI beads to 50 μL of cDNA mix and elute in 33μL of EB solution. Perform SPRI purification by following "**SPRI purification protocol"** shown at the end of this protocol.

End of Day 1, store samples at -20°C. The rest of the protocol offers more opportunities to stop, basically anytime after SPRI purification, store at -20°C and be sure to use non-stick tubes for long term storage.

5. End repair

5.1 Prepare the following reaction mix:

- Eluted DNA 32.4μL
- 10X End-repair buffer 5 μL
- dNTP mix (25mM) 1.6μL
- T4 DNA polymerase (3U/μL) 5μL
- Klenow DNA polymerase (5U/μL) 1μL
- T4 PNK (10U/μL) 5μL

5.2 Incubate at 20°C for 30min.

5.3 Purify the DNA by adding 75 μL of SPRI beads to 50 μL of End-repair-mix and elute in 33μL of EB solution.

6. Adenylation of 3' ends

6.1 Prepare the following reaction mix:

- Eluted DNA 32µL
- "A" Tailing Buffer 5µL
- dATP(1mM) 10µL
- Klenow 3' to 5' exo- (5U/µL) 3µL

6.2 Incubate at 37°C in for 30min.

6.3 Purify the DNA by adding 75 uL of SPRI beads to 50 µL of 'A'tailing-mix and elute in 23µL of EB solution. Perform SPRI purification by following "**SPRI purification protocol"** shown at the end of this protocol.

7. Adaptor ligation

We prefer the T4 DNA ligase available from Enzymatics (and the included 2x rapid buffer) for this step, as with small DNA concentrations, adapter concatimers can be a problem.

7.1 Prepare the following reaction mix:

- Elute DNA 23µL
- DNA rapid 2x ligase buffer 25µL
- PE Adaptor oligo mix (1:15 dilution) 1µL
- T4 DNA ligase (600U/µL) 1µL

7.2 Incubate at RT for 15min.

7.3 Purify the DNA by adding 50 µL of SPRI beads to 50 µL of adaptor-ligation-mix and elute in 30µL of EB solution.

8. PCR

8.1 Set up PCR master mix, make 10% extra reagent for multiple samples, and aliquot 20µL to each PCR tube:

- 5 × cloned Phu Buffer 10µL
- PCR primer 1.0 1µL
- PCR primer 2.0 1µL
- 25mM dNTP mix 0.5µL
- Phu polymerase 0.5µL
- H2O 7µL

8.2 Add 30µL purified ligation mix to the PCR tube.
8.3 Run following PCR cycle:

- 98°C 30 sec
- 98°C 10 sec ←┐
- 65°C 30 sec │ 15×
- 72°C 30 sec ─┘
- 72°C 5 min
- 4°C ∞

8.4 Purify the DNA by adding 50 µL of SPRI beads to 50 µL of PCR product and elute in 15-35µL of EB solution (depending on how small of a sample you started with, and which loading proceedure you plan to use). Perform SPRI purification by following "**SPRI purification protocol**" shown at the end of this protocol.

8.5 Dry down in a vacuum pump for a few minutes, just to make sure all traces of ethanol and other contaminants are removed.

9. Library quantitation

Measure concentration with KAPA Library Quantification Kit (KAPA Biosystems, a qPCR assay, helpful for very low concnentrations) or Agilent bioanalyzer. Reference image from Agilent bioanalyzer.

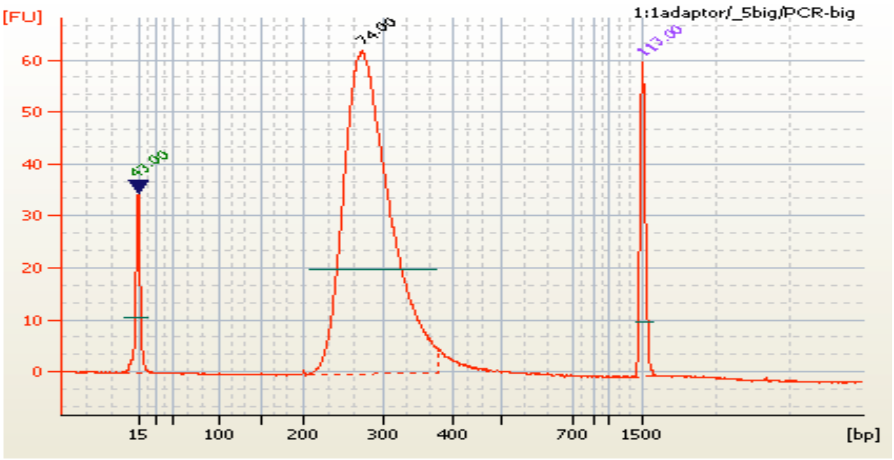

How to perform SPRI (Ampure XP) purification:

1. Transfer *** µL of well mixed SPRI beads (Ampure XP) from stock bottle to each 1.5 mL sample tube.
2. Pipette up and down for at least 8 times for mixing thoroughly.
3. Incubate at room temperature for 5 min.
4. Place 1.5 mL tubes on magnet stand for ~3 min until the liquid appears clear. (Don't rush this step, it's easier to remove the supernatant in the next step without disturbing the beads if you leave it on the magnet a bit longer than you think you should before the next step.
5. Discard the supernatant.
6. Add 180 µL of 70% Ethanol 1.5 mL tube without disturbing the beads. Leave the tubes on the magnet for all wash steps.
7. Wait for 30 seconds and discard the supernatant (70% Ethanol) by using pipette to transfer.
8. Repeat step 6 and 7 for a total of two 70% Ethanol washes.
9. Spin down for 10 seconds at 1000 rpm to spin down any ethanol from side of tube.
10. Place each 1.5 mL tube on magnet stand for 30 seconds then remove all the remaining Ethanol using 10 µl pipette. (There is a delicate balance here of wanting to remove all the ethanol and not wanting to dry the beads too much as this reduces elution efficiency.)
11. Add *** µL of EB to the dry pellet for eluting the DNA. Pipette mixing the pellet and then sit at room temp for 2 min and put on the magnet stand or plate.
12. Transfer clear supernatant to new tubes. Label with all the information and store in - 20 °C.

Materials:

Illumina RNA-seq sample prep kit
Non-Stick RNase-free Microfuge Tubes, 1.5mL (Ambion, AM12450)
Superscript III (Invitrogen, 18080-093) or Superscript II, just adjust PCR program accordingly
Ampure XP beads (Beckman-Coulter, A63880 or A63881)
Magnet stand (we recommend Invitrogen DynaMag-2 , 123-21D)
T4 DNA Ligase, Rapid (Enzymatics, L603-HC-L)
Ethanol
EB buffer
