# Supplementary material for: Noncanonical Compensation of Zygotic X Transcription in Early Drosophila melanogaster Development Revealed through Single-Embryo RNA-Seq
Source: PLoS Biol. 2011 Feb 8;9(2):e1000590. doi: 10.1371/journal.pbio.1000590 (PMC3035605; doi:10.1371/journal.pbio.1000590)
Supplement: Protocol S2 — Low concentration sequencing library loading protocol. (0.08 MB PDF) [file pbio.1000590.s004.pdf]

Low concentration sequencing library loading protocol

(Courtesy of Leath Tonkin of the VCGS Lab, QB3 Institute/UC Berkeley)

For use only if library concentration is well below the requirements (i.e. 1 nM or less) of the Illumina protocol for flowcell loading and cluster station operation. Requires a helpful sequencing facility willing to try something not in the normal protocol. Warning: this is easiest to do if all libraries are of the same volume for the entire flowcell. You should not mix and match libraries of different volumes while attempting this protocol. Using a 20 uL loading volume is the most extreme case, and is only advisable if the library amount is that limited, or if you wish to preserve enough library to run multiple lanes. If at all possible, use a minimum volume of 120 uL and no cluster station protocol modification is required.

Modified Library Denaturation step:
With the Illumina protocol for denaturing and loading of libraries on the flowcell, the amount of final NaOH in the hybridization buffer cannot exceed 8 ul of 0.1 N NaOH in 1 ml of hybridization buffer. This is no problem if the starting library concentration is reasonable, >1 nM. For libraries in the 100's or 10's of pM, a modified approach is needed. We still denature with NaOH, but follow up with an equal amount of HCl to neutralize the pH of the final solution (the astute ones out there will realize this effectively adds NaCl to your library solution

and we don't find that this creates any downstream problems). Assuming you are doing this in the worse case scenario, we provide below an example of how to load a library in a very small volume, 20 uL, since this requires also modifying the cluster station operation. For each library, dilute (or concentrate) 40 pM of library (relative to final volume of solution you are targeting for the lane on the flowcell, a minimum of 20 uL) to 4uL, and add 1 uL of 0.5 N NaOH. Leave libraries at room temperature 5 minutes to denature, then place on ice, then add 1 uL 0.5 N HCl, and dilute to final loading concentration (of at least 20 uL) with cold Illumina hybridization buffer and leave on ice until needed on the cluster station. Fresh dilutions from trusted stock solutions of NaOH and HCl are recommended.

Cluster generation:

Proceed with the appropriate recipe on the cluster station and prepare two (2) of strip tube A (140 uL hybridization buffer) and your 20 uL of denatured library in strip tube B (normally 120 uL). If you have 120 uL of diluted library, the next modified steps on the cluster station are unnecessary and you may proceed with the normal recipe to load and amplify your libraries. To load 20 uL volume libraries on to the flow cell proceed with any recipe that includes the amplification step. Load strip tube A containing hybridization buffer and proceed as directed. The instrument will pause when this step is complete and ask you to load strip tube B containing the 20 ul volume libraries. Do so, but do not put

the end of the hybridization manifold into strip tube B in order to allow a small air gap to form (this will prevent mixing and dilution of the library in the manifold tubing). Press “OK” to start pumping and watch the left end of the hybridization manifold for the air gap to form at the end of all eight lines of tubing. Once there is at least a 3-4 mm gap in all lanes, place the end of the manifold into strip tube B to begin drawing up the libraries into the manifold. Once strip tube B is drained, allow another air gap to form and switch to the second strip tube A containing hybridization solution to fill the remaining volume in the tubing. Watch for the first air gap to move through the flowcell, (right edge) and when the second air gap reaches the front (left edge) of the flowcell, hit the manual stop button on the cluster station software. It is normal to see the air gaps move backwards and the cluster station will take several minutes to pause and become responsive. The idea here is to have the small volume library between the two air gaps and centered within the flowcell. At this point you skip the second “pump reagent” step of the recipe and highlight the “wait” step that follows. Click on the “Start/Resume” button and follow the rest of the recipe with no further modifications. Please see the rudimentary figure for a visual representation of the recipe modifications.

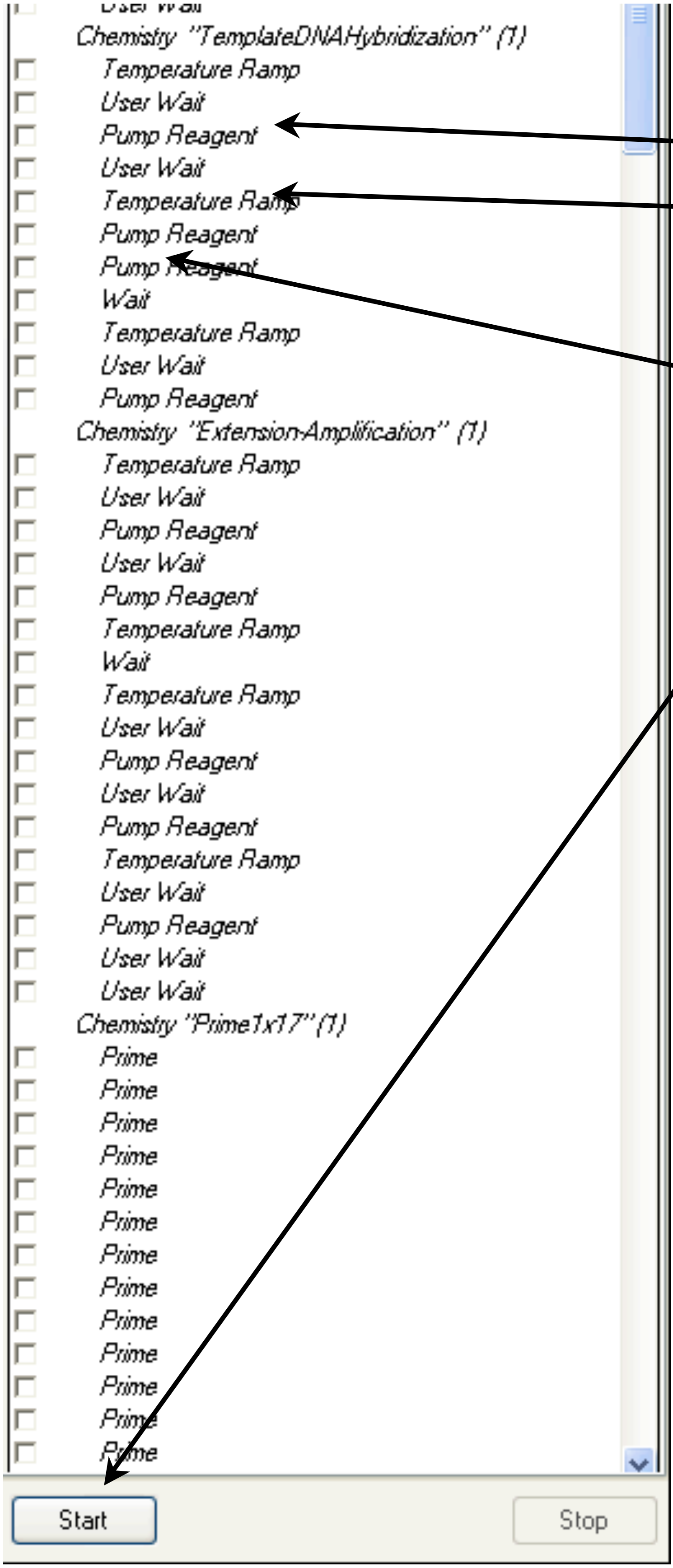

Load strip tube B

You will press "Stop" during this step of the recipe, just before the second air gap enters the flowcell.

When the cluster station has finished entering the safe state, skip over the second "Pump Reagent" and click on the "Wait" step to highlight it.

Finally, click the "Start" button at the bottom to resume the recipe with no further modifications.
